# Longitudinal patterns of regional cerebral blood flow during anti-amyloid therapy in mild cognitive impairment: a pilot ASL MRI case series

**DOI:** 10.3389/fnagi.2026.1903239

**Published:** 2026-09-15

**Authors:** Eva Y. W. Cheung, Yat Fung Shea, Patrick K. C. Chiu, Koon Ho Chan, Hui Zhang, Ka Keung Yam, Kyongtae T. Bae, Henry K. F. Mak

**Affiliations:** 1 Department of Diagnostic Radiology, LKS Faculty of Medicine, The University of Hong Kong, Pokfulam, Hong Kong SAR, China; 2 Division of Geriatrics, Department of Medicine, Queen Mary Hospital, Pokfulam, Hong Kong SAR, China; 3 Department of Rehabilitation Sciences, The Hong Kong Polytechnic University, Kowloon, Hong Kong SAR, China

**Keywords:** amyloid-targeted therapy, anti-amyloid therapy, arterial spin labeling MRI, cerebral blood flow, exploratory neuroimaging, mild cognitive impairment, neurovascular function

## Abstract

**Introduction:**

The neurovascular effects of anti-amyloid therapy remain incompletely understood. Arterial spin labeling (ASL) MRI enables non-invasive quantification of regional cerebral blood flow (rCBF) and may provide insight into perfusion patterns observed over the treatment period.

**Methods:**

This pilot observational case series included participants with MCI or very early Alzheimer’s disease undergoing anti-amyloid therapy (lecanemab), who underwent longitudinal pseudo-continuous ASL MRI at baseline and predefined follow-up timepoints. Regional rCBF was quantified across predefined cortical and subcortical regions of interest. A comparison group of amyloid-positive participants with MCI who did not receive anti-amyloid therapy underwent baseline ASL MRI. Vascular and metabolic risk factors, including white matter hyperintensity burden and biochemical parameters, were assessed to characterize potential confounding influences.

**Results:**

Within the treated group, increased rCBF was observed at early follow-up in several frontal, sensorimotor, and subcortical regions. No statistically significant longitudinal changes were detected at intermediate follow-up. At later follow-up, increased rCBF was observed in the cerebellar cortex, while decreased rCBF was observed in bilateral medial temporal regions. Vascular and metabolic risk profiles, including WMH burden, were broadly comparable between groups.

**Discussion:**

The observed rCBF trajectories demonstrate region-specific and time-dependent perfusion changes over the treatment period. Given the small sample size, absence of longitudinal imaging in the control group, and residual vascular confounding, these findings should be interpreted cautiously as exploratory and hypothesis-generating rather than evidence of treatment efficacy.

## Background

Mild cognitive impairment (MCI) is a heterogeneous clinical syndrome characterized by measurable cognitive decline that does not substantially interfere with daily functioning. While some individuals with MCI progress to Alzheimer’s disease (AD), others remain stable, revert to normal cognition, or progress to non-AD dementias. This heterogeneity complicates early disease staging and monitoring of therapeutic response (Alzheimer’s Association, 2024). With the increasing prevalence of AD and the growing burden on healthcare systems, early intervention in MCI has become a critical focus of research and clinical practice. Among emerging therapeutic strategies, anti-amyloid treatment (AAT) aims to reduce cerebral amyloid burden and has demonstrated disease-modifying effects in subsets of patients with early AD (van Dyck et al., 2023). However, accessible and non-invasive methods for monitoring associated physiological brain changes remain limited. Amyloid positron emission tomography (PET) provides direct molecular assessment (Chételat et al., 2020) but is limited by cost, accessibility, and ionizing radiation. Blood-based biomarkers are emerging as useful screening and monitoring tools but currently offer limited spatial or physiological information.

Arterial Spin Labeling (ASL) MRI enables non-invasive quantification of regional cerebral blood flow (rCBF), serving as an indirect marker of neurovascular function. Prior studies have demonstrated early alterations in rCBF in MCI and AD, reflecting complex interactions between vascular regulation, neuronal activity, and disease progression. However, longitudinal ASL studies examining rCBF changes during anti-amyloid therapy remain scarce.

Recent studies have demonstrated that alternations in CBF are closely associated with amyloid deposition and cognitive decline in MCI and AD (Chau et al., 2020; Korte et al., 2020; Zheng et al., 2019). Regions including posterior cingulate, precuneus and hippocampus – are vulnerable to early amyloid pathology – exhibit perfusion deficits that may precede structural changes. Pseudo-continuous ASL (pcASL) MRI has shown potential in detecting these subtle perfusion changes, making it a promising tool for tracking disease progression and therapeutic response (Alsop et al., 2010; Haller et al., 2016; Alexopoulos et al., 2012).

Despite its advantages, the application of pcASL MRI in monitoring AAT remains underexplored. This study aims to characterize longitudinal and region-specific rCBF changes measured by ASL MRI in individuals with MCI or early AD undergoing anti-amyloid therapy. The objective was to describe temporal perfusion patterns during treatment rather than to establish rCBF as a validated treatment-monitoring biomarker.

## Methods

### Participants

24 participants with mild cognitive impairment (MCI) or early Alzheimer’s disease AD, with reference to the *Revised criteria for diagnosis and staging of Alzheimer’s disease* proposed by the Alzheimer’s Association workgroup (Jack et al., 2024), were recruited from a local memory clinic. Amyloid pathology was confirmed in all participants by 18F-flutemetamol PET imaging. 14 participants in the treated group fulfilled standard clinical eligibility criteria for anti-amyloid therapy (AAT; Lecanemab). 10 control participants were amyloid-positive individuals recruited from the same memory clinic who met diagnostic criteria for MCI but chose not to receive anti-amyloid treatment during the study period.

Eligible participants were adults aged over 55 years. Individuals with active infection; a history of seizures, migraine, psychiatric disorders, significant head injury, or malignancy within the preceding 5 years; or contraindications to MRI (e.g., pacemaker or metallic implants) were excluded. Consistent with clinical safety requirements for anti-amyloid therapy, participants with severe or clinically significant cerebrovascular disease were excluded because of the increased risk of amyloid-related imaging abnormalities (ARIA). Accordingly, individuals with extensive vascular pathology were not eligible for treatment.

However, vascular and metabolic risk factors, including white matter hyperintensity burden and biochemical markers (e.g., glucose and lipid profiles), were assessed to characterize vascular burden and explore potential confounding effects. Despite these assessments, comprehensive vascular characterization and formal statistical adjustment were not performed; therefore, residual confounding cannot be excluded.

### Study design

This was an observational longitudinal study. Participants receiving anti-amyloid therapy underwent ASL MRI at baseline and follow-up timepoints (week 9, week 13, and week 27). Control participants underwent baseline ASL MRI only. Workflow of the study is illustrated in Figure 1.

**Figure 1:**
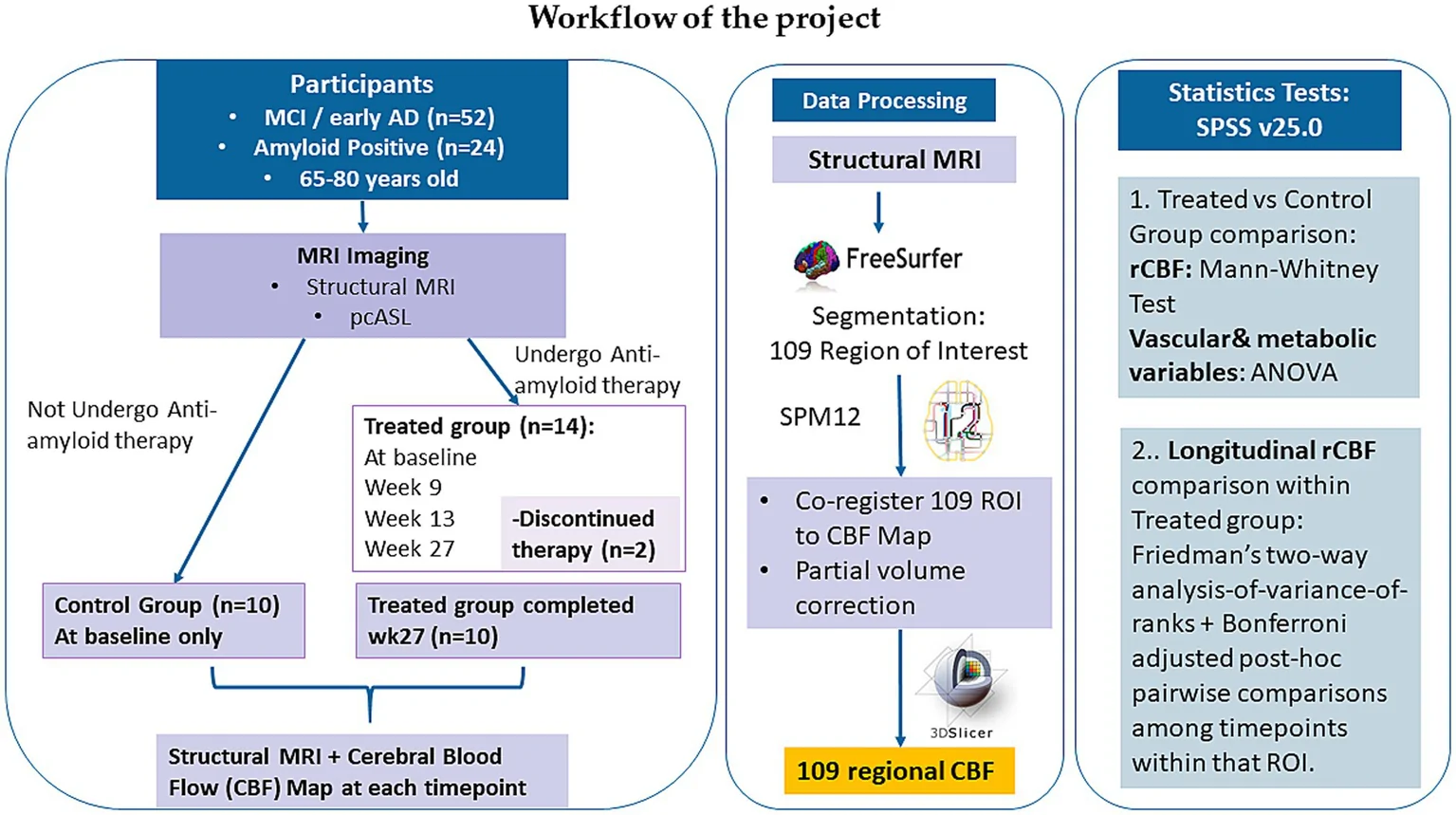
Workflow of the study.

### Imaging acquisition and processing

All MRI examinations were performed on the same 3 T GE SIGNA Premier scanner. T1-weighted 3D Magnetization-prepared-Rapid-Gradient-echo (MPRAGE) was acquired for anatomical reference. Pseudo-continuous arterial spin labeling (pcASL) imaging was performed in accordance with established clinical ASL recommendations of the ISMRM Perfusion Study Group and the European Consortium for ASL in Dementia (Alsop et al., 2015). The acquisition protocol included labeling duration of 1,500 ms. An initial post-label delay (PLD) of 2025 ms was selected to accommodate potentially prolonged arterial transit times commonly observed in older adults and individuals with cognitive impairment. When globally low cerebral blood flow values (<30 mL/100 g/min across the majority of brain regions) were observed, a second pCASL acquisition was performed using a longer PLD of 2,525 ms to reduce potential underestimation of cerebral blood flow resulting from delayed label arrival.

The selected PLDs were based on our previous experience in dementia cohorts, in which these delay times were found to provide adequate perfusion measurements in the majority of patients while maintaining a clinically acceptable acquisition time. Additional intermediate PLDs were therefore not routinely acquired, as the protocol was designed primarily for clinical perfusion assessment rather than comprehensive arterial transit time characterization. A 3D readout scheme with background suppression pulses was used.

Quantitative cerebral blood flow (CBF) maps were generated using the vendor-implemented absolute quantification model available on the 3 T GE SIGNA Premier platform. This implementation is based on the consensus single-compartment kinetic model described in the ISMRM/European ASL White Paper recommendations and incorporates standard physiological assumptions for ASL perfusion quantification. CBF estimation was performed using the corresponding proton density reference image provided by the acquisition protocol. Total acquisition time was approximately 4.5 min. Arterial transit time was not estimated in the present study because the clinical protocol was designed for routine CBF quantification rather than ATT mapping.

Partial Volume Correction (PVC) was performed using built-in SPM toolbox PETPVE12. Although originally developed for PET applications, the geometric transfer matrix approach has also been applied in cerebral perfusion imaging studies and was used here to reduce tissue-mixing effects in regional CBF estimates.

Image processing included motion correction using SPM and tissue segmentation using FreeSurfer (Fischl, 2012). The CBF maps from each timepoint were co-registered to the corresponding T1-weighted anatomical images. ROI-based rCBF measurements were extracted using 3D slicer software (The Slicer Community; V.4.11.20210226) with the PyRadiomics extension. Detail acquisition parameters and imaging processing procedures were provided in Supplementary materials.

### Statistical analysis

Given the small sample size and non-normal distributions, non-parametric statistical tests were applied. rCBF of all participants were analyzed using SPSS software version 25.0 (SPSS Inc., Chicago, IL, United States).

Treated group and control group comparison: The means of rCBF obtained within the 109 ROIs was compared by Mann–Whitney U test. Vascular and metabolic variables, including fasting glucose, HbA1c, lipid profile parameters, and volumetric white matter hyperintensity (WMH) burden, were compared between treated and control groups using one-way analysis of variance (ANOVA). These analyses were descriptive and performed to assess potential group differences in vascular risk profiles. Due to the limited sample size, analyses of vascular and metabolic variables were conducted descriptively without further multivariable adjustment.

Longitudinal rCBF comparison within Treated group were assessed using Friedman’s test separately for each ROI across four timepoints. When a significant Friedman test was observed, Bonferroni adjusted post-hoc pairwise comparisons were performed to account for multiple comparisons among timepoints within that ROI. Adjusted *p*-values *p* < 0.05 were considered statistically significant.

Given the exploratory nature of this pilot case series, correction was applied to pairwise temporal comparisons within each ROI rather than across all 109 ROIs. Findings should therefore be interpreted as hypothesis-generating observations.

The 109 ROIs were listed in Supplementary Table A.

## Results

Between July 2024 and March 2025, 52 participants were screened, and 14 individuals with MCI were prescribed anti-amyloid therapy. Two participants discontinued therapy due to amyloid-related imaging abnormalities (they developed ARIA after 4th dose, and after 6th dose respectively), and ten participants completed follow-up through week 27.

The treated group consisted of three men and seven women, aged between 66 and 87 years, with a mean baseline Montreal Cognitive Assessment (Hong Kong version) score of 18.37 ± 2.66, and a mean amyloid burden of 77.03 ± 21.24 Centiloids. Amyloid clearance values were not available because treatment was ongoing at the time of analysis.

The control group comprised ten amyloid-positive participants with MCI (five men and five women), aged between 58 and 82 years, with a mean Montreal Cognitive Assessment score of 18.00 ± 5.62 and a mean amyloid burden of 58.80 ± 36.35 Centiloids.

Participant demographics are summarized in Table 1.

**Table 1:** Participant demographics (treated vs. control).

Participant Characteristics	Treated Group	Control group	*p*-value
Age	66–87	58–82	0.556
Sex (M:F)	3:7	5:5	0.13
HK-MoCa score	18.37 ± 2.66	18.00 ± 5.62	0.87
Centiloid level	77.03 ± 21.24	58.80 ± 36.35	0.33

Vascular and metabolic risk factors were assessed to evaluate potential confounding effects. There were no statistically significant differences between the treated and control groups in fasting glucose, HbA1c, lipid profile parameters including total cholesterol, triglycerides, HDL-C, LDL-C, and non-HDL-C. These findings suggest that overall vascular and metabolic risk profiles were broadly comparable between groups.

Volume WMH burden was comparable between treated and control groups, suggesting that major differences in small-vessel disease burden are unlikely to explain the observed perfusion patterns. Nevertheless, the presence of residual vascular confounding cannot be excluded. Details of results were listed in Table 2.

**Table 2:** Participant vascular characteristics (treated vs. control).

Participant vascular characteristic	Treated group	Control group	*p*-value
Fasting glucose (mmol/L)	6.733 ± 1.80	5.20 ± 0.95	0.158
HbA1c (%)	8.63 ± 4.66	6.12 ± 0.72	0.324
Total cholesterol	4.22 ± 0.49	5.10 ± 2.08	0.383
Triglycerides	1.76 ± 0.69	0.95 ± 0.56	0.051
HDL-C	1.23 ± 0.21	1.77 ± 0.53	0.062
LDL-C	2.13 ± 0.31	2.89 ± 1.78	0.376
Non-HDL-C	2.89 ± 0.51	3.33 ± 1.68	0.588
WMH volume (mm^3^)	3671.22 ± 3652.7	5268.98 ± 4905.2	0.468

### Overview of longitudinal rCBF changes

Regional cerebral blood flow changes varied by brain region and timepoint. At early follow-up (week 9), increased rCBF was observed across multiple frontal, sensorimotor, and subcortical regions. These changes were not sustained at the intermediate follow-up (week 13), where no statistically significant differences were detected. At later follow-up (week 27), increased rCBF was observed in the cerebellar cortex, whereas decreased rCBF was observed in medial temporal regions, including the bilateral entorhinal and para-hippocampal gyri (Figure 2).

**Figure 2:**
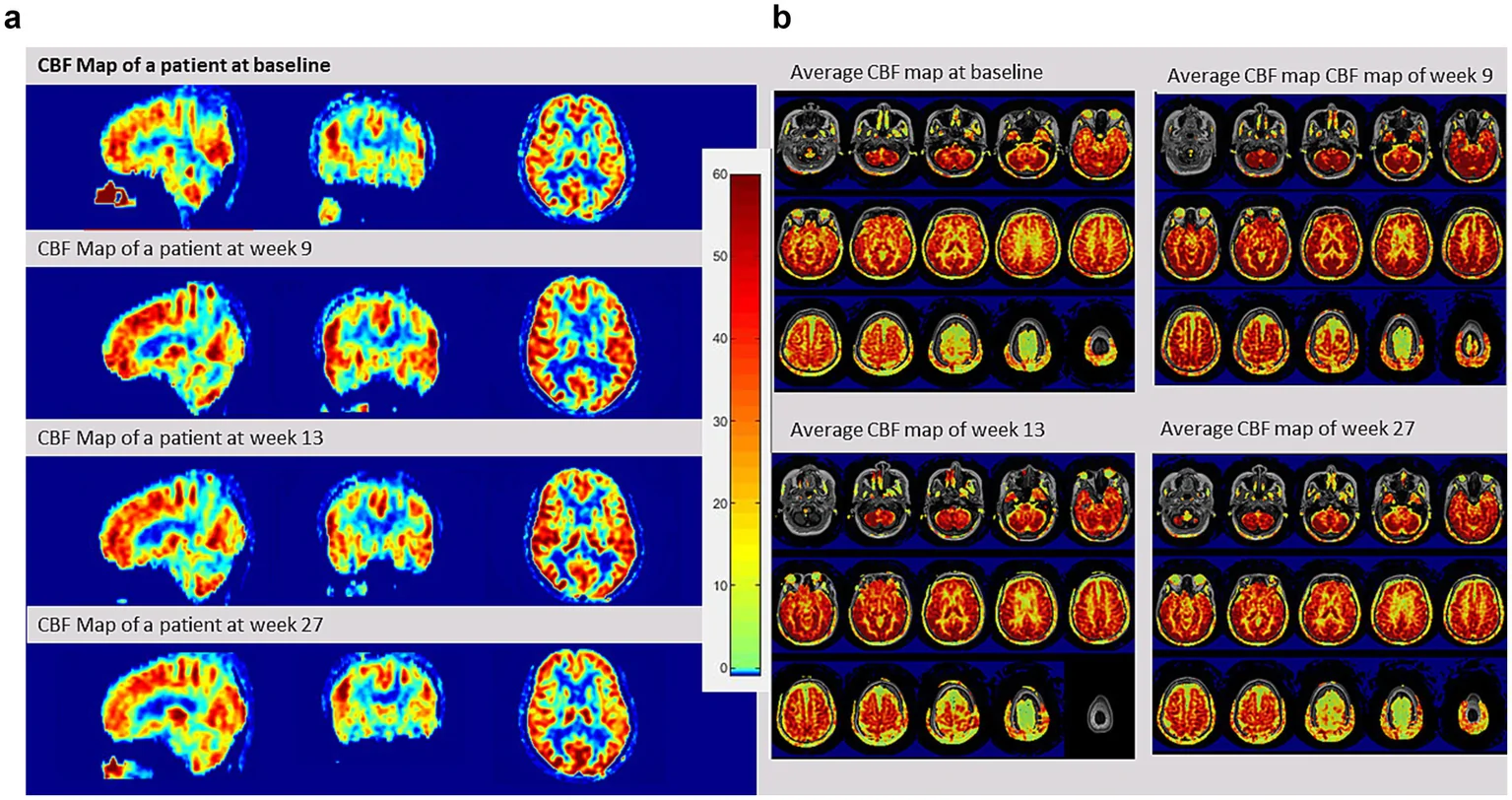
CBF map of participants in the treated group at various time points during the treatment. **(a)** Representative CBF maps from a single participant in the treated group at baseline, week 9, week 13, and week 27. Images are presented for visualization purposes only and are not intended for quantitative comparison. **(b)** Average CBF map of all patients from treated group at baseline, week 9, week 13, and week 27.

### Baseline comparison between treated and control groups

No statistically significant differences in rCBF were observed between the treated and control groups at baseline.

### rCBF changes at early follow-up (week 9) (all in units ml/100 g/min)

At early follow-up (week 9), increased rCBF was observed across multiple subcortical and cortical regions.

Subcortical regions showing increased rCBF including left pallidum (33.8 ± 8.9 to 35.20 ± 7.22,), right amygdala (36.75 ± 8.59 to 40.29 ± 14.22,), and right nucleus accumbens (44.20 ± 7.59 to 47.42 ± 12.72, all *p* < 0.05).

Cortical regions with increased rCBF were predominantly located in frontal and sensorimotor areas, including the bilateral paracentral gyri (left: 42.73 ± 7.47 to 47.54 ± 8.24; right: 43.72 ± 7.18 to 48.42 ± 9.06), left precentral gyrus (40.72 ± 5.67 to 44.95 ± 7.53), left rostral middle frontal gyrus (42.90 ± 5.78 to 48.34 ± 12.46), left superior frontal gyrus (40.74 ± 5.45 to 44.66 ± 11.43), frontal pole (41.11 ± 3.76 to 50.06 ± 11.38), right pars triangularis gyrus (47.01 ± 7.92 to 51.85 ± 10.64), right postcentral gyrus (42.70 ± 4.53 to 46.95 ± 7.90), and right supramarginal gyrus (42.92 ± 6.36 to 49.31 ± 11.50) (all *p* < 0.05).

### rCBF changes at intermediate follow-up (week 13)

No statistically significant differences in rCBF were observed between baseline and week 13.

### rCBF changes at later follow-up (week 27)

At week 27, increased rCBF was observed in the left cerebellar cortex (42.19 ± 10.15 to 45.94 ± 7.97, *p* = 0.025).

In contrast, decreased rCBF was observed in medial temporal regions, including the bilateral entorhinal gyri (left: 33.45 ± 4.90 to 26.35 ± 6.80, *p* = 0.025; right: 34.48 ± 8.75 to 29.15 ± 7.43, *p* = 0.041) and bilateral parahippocampal gyri (left: 41.71 ± 6.08 to 34.36 ± 6.67, *p* = 0.026; right: 45.68 ± 8.12 to 39.35 ± 11.42, *p* = 0.034).

Longitudinal rCBF trajectories across the left and right hemispheres are illustrated in Figures 3a,b, respectively. Regional distributions of increased rCBF at week 9 and decreased rCBF at week 27 are shown in Figures 4a,b.

**Figure 3:**
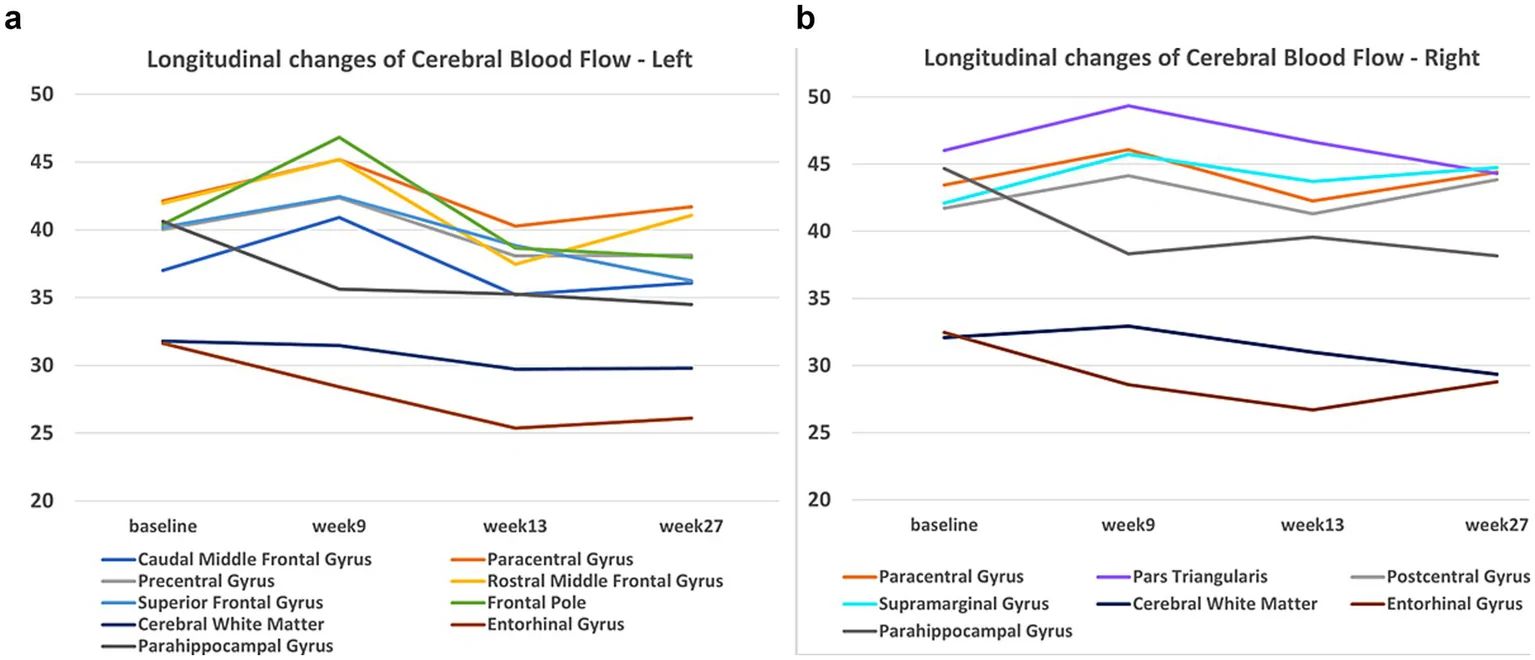
For visualization clarity, only selected representative regions demonstrating statistically significant longitudinal changes are shown. **(a)** Left Hemisphere regional cerebral blood flow trajectories. Longitudinal changes in mean regional cerebral blood flow (rCBF) across selected left-hemispheric cortical and subcortical regions at baseline, week 9, week 13, and week 27 in the treated group. Values are shown in mL/100 g/min and illustrate relative temporal patterns rather than inferential comparisons. **(b)** Right hemisphere regional cerebral blood flow trajectories. Longitudinal changes in mean regional cerebral blood flow (rCBF) across selected right-hemispheric cortical and subcortical regions at baseline, week 9, week 13, and week 27 in the treated group. Shown regions represent those demonstrating statistically significant or notable longitudinal changes.

**Figure 4:**
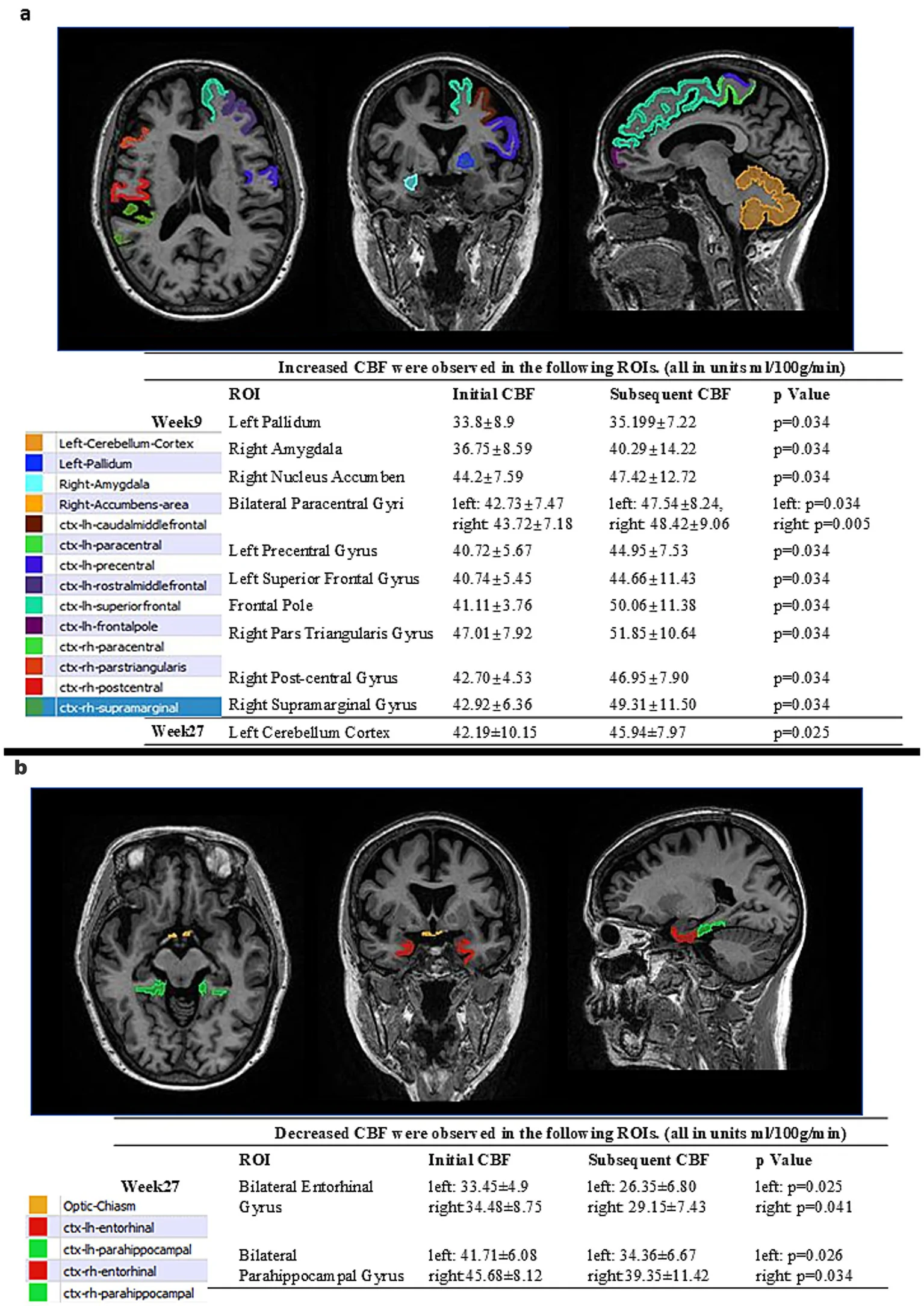
Regions demonstrating changes in rCBF at various timepoints relative to baseline. **(a)** Regions demonstrating increased rCBF at week 9 relative to baseline. Representative atlas-based overlays illustrating cortical and subcortical regions showing increased regional cerebral blood flow (rCBF) at early follow-up (week 9) relative to baseline in the treated group. Corresponding summary statistics are shown below. Images are presented for visualization purposes only. **(b)** Regions demonstrating decreased rCBF at week 27 relative to baseline. Representative atlas-based overlays illustrating regions showing decreased regional cerebral blood flow (rCBF) at later follow-up (week 27) relative to baseline. Statistical comparisons for bilateral entorhinal, parahippocampal, and other regions are summarized below. Visualizations are illustrative and not intended for voxel-wise interpretation.

When considering mean cortical gray matter rCBF, several participants exhibited increasing cortical perfusion values during follow-up. The magnitude and direction of changes varied considerably among individuals. These findings further support the heterogeneous nature of perfusion responses observed in this case series. Figure 5 presents participant-level plots of mean cortical gray matter rCBF across all four timepoints, allowing visualization of inter-individual variability throughout the treatment period.

**Figure 5:**
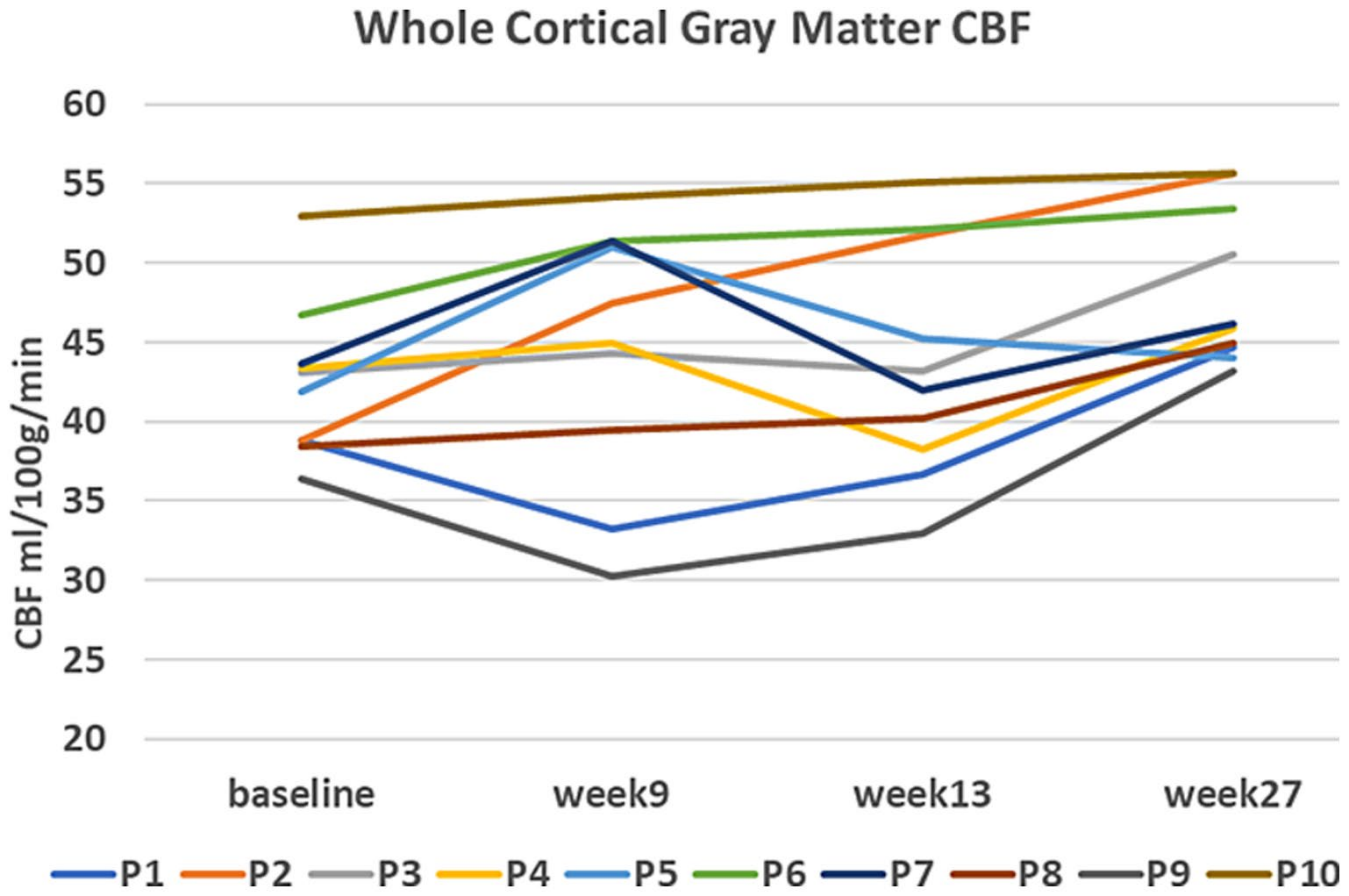
Participant-level plots of mean cortical gray matter rCBF across all four timepoints, allowing visualization of inter-individual variability throughout the treatment period.

### ARIA cases

Two participants developed amyloid-related imaging abnormalities (ARIA) during anti-amyloid therapy and subsequently discontinued treatment. Because these participants did not complete the predefined longitudinal imaging protocol through week 27, they were excluded from the primary longitudinal analyses. Nevertheless, their imaging findings are presented descriptively to provide additional context regarding treatment-related adverse events.

Participant 1 developed moderate ARIA-E and ARIA-H after the fourth treatment dose. FLAIR imaging demonstrated multifocal vasogenic edema involving several cerebral regions, consistent with moderate ARIA-E (>1 site of edema). Corresponding susceptibility-weighted imaging (SWI) demonstrated multiple cerebral microbleeds, consistent with moderate ARIA-H (≥5 microbleeds). Corresponding ASL perfusion maps suggested reduced perfusion within regions affected by vasogenic edema and microbleeds (Figure 6a). However, interpretation should be made cautiously because local mass effect and tissue displacement associated with edema may influence the appearance of the perfusion maps.

**Figure 6:**
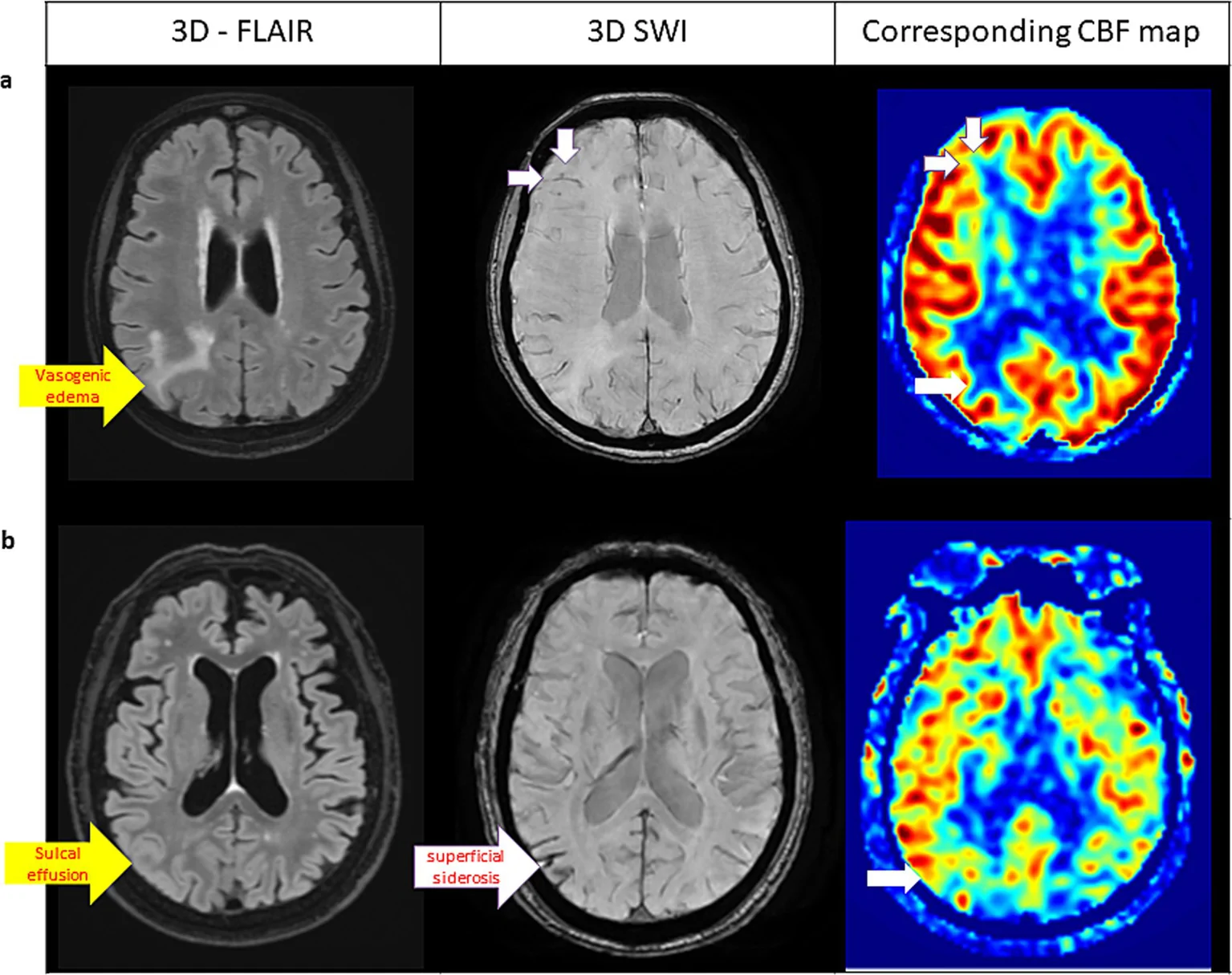
Imaging findings in participants who developed amyloid-related imaging abnormalities (ARIA). **(a)** Participant 1. Axial FLAIR images demonstrate multifocal vasogenic edema consistent with moderate ARIA-E. Corresponding SWI images demonstrate multiple cerebral microbleeds consistent with moderate ARIA-H. Corresponding ASL cerebral blood flow maps suggest reduced perfusion within regions affected by vasogenic edema. These findings are descriptive and should be interpreted cautiously. **(b)** Participant 2. Axial FLAIR images demonstrate a focal right lateral occipital sulcal effusion measuring approximately 0.6 cm, consistent with mild ARIA-E. Corresponding SWI images demonstrate adjacent cortical superficial siderosis consistent with ARIA-H. Corresponding ASL cerebral blood flow maps are shown for descriptive purposes only. Owing to limited image quality, no reliable interpretation regarding regional perfusion changes could be made. These participants discontinued anti-amyloid therapy before completion of the predefined longitudinal imaging protocol and were therefore excluded from the primary longitudinal statistical analyses.

Participant 2 developed mild ARIA-E and ARIA-H after the sixth treatment dose. FLAIR imaging demonstrated a focal right lateral occipital sulcal effusion measuring approximately 0.6 cm, without associated parenchymal vasogenic edema. SWI demonstrated a thin curvilinear focus of cortical superficial siderosis adjacent to the sulcal effusion, consistent with ARIA-H. Corresponding ASL cerebral blood flow maps are shown for reference (Figure 6b). Owing to limited image quality, no reliable conclusion regarding regional perfusion changes could be drawn in this case.

Because only two ARIA cases were identified, no formal statistical analysis was performed and no conclusions regarding the predictive value of ASL findings can be drawn.

## Discussion

This exploratory case series identified heterogeneous, region-specific, and time-dependent rCBF changes in individuals with MCI or early AD undergoing anti-amyloid therapy. The longitudinal trajectories observed in Figure 2 highlight that rCBF changes during anti-amyloid therapy are not uniform or monotonic, but vary by region and timepoint, underscoring the dynamic and heterogeneous nature of neurovascular response. Increased CBF was observed in multiple cortical and subcortical regions including the pallidum, right amygdala, nucleus accumbens, paracentral and precentral gyri, frontal gyrus and cortex, pars triangularis, postcentral gyrus, supramarginal gyrus and the cerebellum cortex. These regions are functionally associated with motor control, emotion regulation, executive processing and sensory integration. These findings may reflect transient neurovascular or functional adaptations occurring during treatment, although alternative explanations such as physiological variability or disease progression cannot be excluded. Amyloid-β is known to impair endothelial function and constrict cerebral capillaries by pericyte-mediated mechanisms, leading to hypoperfusion in affected regions (Zhang et al., 2012). These observations may reflect transient neurovascular or physiological adaptations occurring over the treatment period (Iturria-Medina et al., 2016); however, causal attribution to anti-amyloid therapy or amyloid clearance cannot be established. The cerebellum cortex, traditionally associated with motor coordination, has been recognized for its role in cognitive and affective processing. Increased perfusion in these regions was observed; however, the functional significance of these changes remains uncertain. A recent study suggested that the cerebellum participates in distributed cognitive networks and may support executive and memory functions in neurodegenerative conditions (Schmahmann, 2019). Enhanced perfusion in the frontal, sensorimotor and subcortical regions at early follow-up may reflect transient neurovascular or functional adaptations during treatment. The absence of detectable changes at intermediate follow-up (week 13) may be related to physiological variability or limited statistical power. Increased cerebellar perfusion at later follow-up is consistent with emerging evidence that the cerebellum participates in distributed cognitive networks and may demonstrate compensatory engagement in neurodegenerative conditions, however, causal attribution to amyloid clearance cannot be established.

Conversely, decreased CBF was detected in the bilateral entorhinal and parahippocampal gyri, which are critically involved in episodic memory and among the earliest affected by AD pathology. These areas are particularly vulnerable to MCI and AD neurodegenerations, which may persist despite reductions in amyloid burden (Graff et al., 2023). Structural and functional imaging studies have consistently shown that medial temporal lobe regions exhibit early atrophy and hypoperfusion in both MCI and AD, often correlating with cognitive decline (Zheng et al., 2019; Sierra-Marcos, 2017). The sustained reduction in CBF in these regions may reflect irreversible neuronal loss, impaired neurovascular coupling, or microvascular damage that is not fully reversible with anti-amyloid therapy alone.

Several regions demonstrating longitudinal perfusion changes in the present study correspond to areas known to be vulnerable to Alzheimer’s disease pathology. In particular, the entorhinal and parahippocampal cortices are among the earliest regions affected by amyloid accumulation and subsequent neurodegeneration (Harris et al., 2010). The observation that perfusion changes occurred within these anatomically susceptible regions supports the biological plausibility of the findings. However, direct linkage between regional perfusion changes and underlying pathological processes cannot be established in the absence of longitudinal molecular biomarker measurements.

The differential CBF responses observed across brain regions suggested that while some areas may exhibit transient increases in perfusion, although sustained functional recovery cannot be inferred from the present data, others may continue to decline due to underlying pathology. These findings are consistent with previous reports of heterogeneous and temporally variable perfusion patterns observed in similar populations (Korte et al., 2020; Zheng et al., 2019; Zhang et al., 2021).

Recent studies using ASL MRI have reported region-specific and temporally variable CBF changes in patients undergoing anti-amyloid therapy. In a recent ASL case series of patients treated with Lecanemab, longitudinal perfusion changes were heterogeneous, with some individuals showing early regional increases in cerebral blood flow, others demonstrating transient changes, and some showing no measurable perfusion response despite ongoing therapy. Importantly, medial temporal regions were not uniformly responsive, highlighting that amyloid reduction does not necessarily translate into normalization of regional perfusion. Complementary ASL studies in MCI and early Alzheimer’s disease populations have similarly demonstrated persistent hypoperfusion and disrupted perfusion networks in entorhinal and hippocampal regions, underscoring their early and sustained vulnerability to neurodegenerative processes (Ricaurte-Fajardo et al., 2025; Li et al., 2024). In this context, the present findings are consistent with emerging evidence that cerebral perfusion responses during anti-amyloid therapy are heterogeneous and likely reflect a combination of transient neurovascular adaptation, compensatory network engagement, and ongoing neurodegeneration rather than a uniform treatment effect. However, in the absence of tau or other molecular biomarkers, the underlying pathological drivers of these changes cannot be determined.

The absence of significant differences in vascular and metabolic risk factors between groups, including quantitative WMH measures and lipid and glycemic parameters, suggests that these factors are unlikely to fully account for the observed rCBF patterns, although residual confounding cannot be excluded.

An alternative explanation for some of the observed perfusion changes is a transient vascular or inflammatory response associated with antibody-mediated amyloid engagement. Because inflammatory biomarkers and longitudinal vascular imaging markers were not collected, the present dataset cannot distinguish among these potential mechanisms.

Importantly, interpretation is limited by the absence of longitudinal imaging in the control group. Observed rCBF changes may reflect natural disease progression, physiological variability, or treatment-associated effects. Accordingly, the present findings should be regarded as hypothesis-generating.

Although only two participants developed ARIA, the corresponding ASL perfusion maps provided descriptive physiological information within regions affected by ARIA-related changes. Because of the limited number of cases and image-quality limitations, no meaningful conclusions regarding perfusion abnormalities, ARIA prediction or ARIA monitoring can be drawn. Nevertheless, these observations highlight a potential area for future investigation in larger prospective studies.

The present study was not designed to evaluate treatment effects but to describe temporal perfusion patterns in a real-world clinical setting.

### Limitations

The present study should be regarded as a pilot descriptive case series intended to characterize temporal perfusion patterns observed over the treatment period rather than to evaluate treatment efficacy. Owing to the absence of longitudinal control imaging and the limited sample size, the findings should be interpreted as hypothesis-generating observations.

This study has several limitations. The sample size remains small relative to the number of regions analyzed, limiting statistical power and increasing susceptibility to variability. Although vascular and metabolic risk factors—including WMH burden and biochemical markers—were assessed descriptively, formal statistical adjustment was not performed, and residual confounding cannot be excluded. While participants with severe cerebrovascular disease were excluded based on clinical criteria, subclinical vascular pathology may still have influenced ASL-derived rCBF measurements.

In addition, the study did not include tau or cerebrospinal fluid biomarkers, limiting the ability to directly relate perfusion changes to tau pathology. Control participants did not undergo longitudinal imaging, restricting causal inference and limiting differentiation between treatment-associated effects, disease progression, and physiological variability. Finally, molecular biomarkers and detailed cognitive–imaging correlations were not examined. Because multiple ROIs were examined in a small cohort, the observed regional findings require confirmation in larger longitudinal studies and should be considered exploratory. Future studies incorporating larger cohorts, systematic vascular characterization, multimodal molecular biomarkers, repeated longitudinal imaging in control participants, and extended follow-up will be necessary to further clarify the role of rCBF changes during anti-amyloid therapy.

### Implication for practices

ASL MRI may provide physiological insight into neurovascular changes occurring during anti-amyloid therapy. The observed regional and time-dependent perfusion patterns suggest heterogeneous neurovascular responses rather than uniform recovery. While ASL is not suitable as a standalone treatment-monitoring biomarker, it may contribute to future multimodal frameworks integrating imaging with fluid biomarkers.

## Conclusion

Rather than indicating a consistent or sustained treatment effect, the observed rCBF trajectories suggest temporally and regionally heterogeneous physiological changes during anti-amyloid therapy. These findings highlight the dynamic nature of cerebral perfusion in this population and underscore the complexity of interpreting rCBF changes in the context of disease progression and treatment exposure. While ASL MRI may provide exploratory insight into neurovascular dynamics during therapy, the present results should be interpreted cautiously and do not constitute evidence of treatment efficacy nor validation of ASL-derived rCBF as a treatment-monitoring biomarker. Confirmation of clinical relevance will require larger, well-controlled studies incorporating systematic vascular characterization, longitudinal control data, and multimodal biomarkers.

## Data Availability

The raw data supporting the conclusions of this article will be made available by the authors, without undue reservation.
