# Supplementary material for: Longitudinal patterns of regional cerebral blood flow during anti-amyloid therapy in mild cognitive impairment: a pilot ASL MRI case series
Source: Front Aging Neurosci. 2026 Sep 15;18:1903239. doi: 10.3389/fnagi.2026.1903239 (PMC13619891; doi:10.3389/fnagi.2026.1903239)
Supplement: Supplementary file 1 [file Supplementary_file_1.docx]

**Supplementary file**

1. **Image Acquisition protocol**

**Structural MRI:** Anatomical reference and co-registration.

Image acquisition: 3D T1-weighted MPRAGE images will be obtained using the following parameters: repetition time (TR)=7.3ms, echo time (TE)=2.94ms, inversion time (TI)=900ms, flip angle=8°, slice thickness=1mm, 340 sagittal slices, acquisition matrix=256×256, field of view (FOV)=256×256mm^2^, voxel size=1×1×1mm^3^, band width=122 Hz/pixel.

**pcASL:** regional CBF

Image acquisition: Images will be obtained using the following parameters: Look-locker based echo planar imaging sequence, 3D readout scheme, background suppression pulses, and acquisition of a proton density reference image for CBF quantification, TR/TE=5415/53ms, Labelling duration: 1500ms; Post Label Delay (PLD)= 2025ms to 2525ms; FOV=240×240mm^2^, slice-thickness=4mm, slices-spacing=4mm, acquisition matrix =512×512mm^2^.

Prior to routine system updates: TR/TE=5410/52ms;

2. **Image processing steps**
a. FreeSurfer segmentation procedures and algorithms employed were documented on the website http://surfer.nmr.mgh.harvard.edu/
b. Pre-processing of images included motion correction, spatial normalization to standard MNI space, and smoothing were performed with Statistical Parametric Mapping software version 12 (SPM12) ^11^. Partial volume correction (PVC) was performed using the PETPVE12 toolbox implemented in SPM. Although originally developed for PET applications, the geometric transfer matrix approach has also been applied in cerebral perfusion imaging studies and was used here to reduce tissue-mixing effects in regional CBF estimates. FreeSurfer v7.1.0 image analysis suite was employed to perform brain segmentation for 41 segmented subcortical structures with reference to Automated Anatomical Labeling version 3 (AAL3) brain atlas, and cortical parcellation with reference to Desikan-Killiany atlas in MNI space ^12^ for 68 parcellated cortical gyri and sulci^13^. Further, 3D slicer software (The Slicer Community; V.4.11.20210226) with the PyRadiomics extension was employed for the rCBF extraction^14^.
c. Details of ROIs were shown in table A.

Table A showed the 109 regions of interested: 41 segmented subcortical structures with reference to Automated Anatomical Labelling version 3 (AAL3) brain atlas, and cortical parcellation with reference to Desikan-Killiany atlas in MNI space for 68 parcellated cortical gyri and sulci were obtained. (total 109 ROIs were obtained).

| **109 Regions of Interests** | | | |
| --- | --- | --- | --- |
| **41 Segmented subcortical structures** | | **68 Parcellated cortical gyri and sulci** | |
| **11 ROI** | **Left & Right side (15 ROI each)** | **Left & Right side: 34 ROI each** | |
| Third Ventricle | Cerebral-White-Matter | Bankssts | Pars orbitalis |
| Fourth Ventricle | Lateral-Ventricle | Caudal anterior cingulate | Pars triangularis |
| Brain Stem | Inf-Lat-Vent | Caudal middle frontal | Pericalcarine |
| CSF | Cerebellum-White-Matter | Cuneus | Postcentral |
| WM hypointensities | Cerebellum Cortex | Entorhinal | Posterior cingulate |
| Optic Chiasm | Thalamus | Fusiform | Precentral |
| CC_Posterior | Caudate | Inferior parietal | Precuneus |
| CC_Mid_Posterior | Putamen | Inferior temporal | Rostral anterior cingulate |
| CC_Central | Pallidum | Isthmus cingulate | Rostral middle frontal |
| CC_Mid_Anterior | Hippocampus | Lateral occipital | Superior frontal |
| CC_Anterior | Amygdala | Lateral orbitofrontal | Superior parietal |
|  | Accumbens area | Lingual | Superior temporal |
|  | Ventral DC | Medial orbitofrontal | Supramarginal |
|  | Vessel | Middle temporal | Frontal pole |
|  | Choroid plexus | Para hippocampal | Temporal pole |
|  |  | Paracentral | Transverse temporal |
|  |  | Parsopercularis | Insula |
